# Reasons for using cannabidiol: a cross-sectional study of French cannabidiol users

**DOI:** 10.1186/s42238-021-00102-z

**Published:** 2021-10-06

**Authors:** Davide Fortin, Vincent Di Beo, Sophie Massin, Yann Bisiou, Patrizia Carrieri, Tangui Barré

**Affiliations:** 1 University Paris 1 Sorbonne, Paris, France; 2 Aix Marseille Univ, Inserm, IRD, SESSTIM, Sciences Economiques & Sociales de la Santé & Traitement de l’Information Médicale, ISSPAM, Marseille, France; 3 Univ. Artois, CNRS, IESEG School of management, Univ. Lille, UMR 9221, Lille Economie Management (LEM), F-62000 Arras, France; 4 University Paul Valéry Montpellier 3, CORHIS , Montpellier, France

**Keywords:** Cannabidiol, France, Well-being, Cannabis, Stress

## Abstract

**Background:**

Cannabidiol and cannabidiol-based products are proliferating in many countries. This recent and rapid diffusion prompts investigating the reasons for its use.

**Methods:**

We analyzed data from an online survey among cannabidiol users in the French general population (*n* = 1166) selected for their interest in such products. We described the reported reasons for using cannabidiol. We performed logistic regressions to identify the correlates of declaring well-being and other specific reasons for using cannabidiol. We also provided descriptive data regarding the cannabidiol patterns of use.

**Results:**

Well-being was the most cited primary reason for use (27% of the sample). Declaring well-being as a primary reason for using cannabidiol was inversely associated with cigarette smoking, cannabis use, and employment. Among cannabidiol users reporting well-being as their primary reason for use, stress and sleep improvements were the most-cited specific reasons. In the whole study sample, the most common modes of use were smoking cannabidiol-rich cannabis (61%) and ingesting cannabidiol oil sublingually (19%).

**Conclusions:**

In a sample of cannabidiol users from France, well-being was the most-cited primary reason for use, and smoking was the first route of administration. Further research is needed to clarify to what extent expected effects are scientifically sound and to understand country-related specificities regarding patterns of use.

## Introduction

Cannabidiol (CBD)-based products are proliferating in countries with different legislation regarding the use of cannabis-based products (Walker et al., 2020). Concerns about the safety of CBD-based products have been raised (Lachenmeier et al., 2019). Given the high risk of misleading information and subsequent confusion about CBD’s legal status and its effects, there is a need to elucidate motivations and patterns of use among CBD users. Recently, in the UK, Moltke et al. investigated the reasons for CBD use and patterns of use among 387 CBD users (Moltke & Hindocha, 2021). Using an online questionnaire, they found that self-perceived anxiety, sleep problems, stress, and general health and well-being were the top 4 reasons for CBD use. Moreover, female users were less likely to declare general health and well-being as a reason for CBD use, and sublingual administration was by far the most-cited route of CBD administration.

By using data from an online survey among CBD users, we aimed to identify sociodemographic and behavioral correlates of using CBD for general well-being and more specific purposes in French users, as well as present associated patterns of use. By doing so, we aimed at replicating Moltke et al.’s investigations and potentially highlight country-specific differences.

## Material and methods

Using a design quite similar to that of Moltke et al., we conducted an anonymous online survey among CBD users mostly living in France. The protocol followed the guidelines of the Declaration of Helsinki, and the INSERM Ethics Committee provided ethical approval (approval #20-677 dated April 23, 2020). An online link to the survey was distributed via media outlets specializing in cannabis-based products, CBD user groups on Facebook, and a community of people with chronic health conditions. Therefore, participants were enrolled from the general population. Data were collected using a Google survey form between 23 April 2020 and 30 March 2021. In the survey, “CBD” included all legal CBD-rich products, including CBD-rich cannabis. Certain questions regarded patterns of use and collected, among other information, time since first CBD use, number of days CBD was used in the previous 30 days, time of day when CBD was used, CBD purchase locations in the previous 30 days, and principal type of CBD product used over the same period. Another question asked about the respondents’ primary reason for using CBD in the previous 30 days (only one answer possible) with several options regarding well-being, psychoactive substance use reduction, curiosity, and socialization. People who answered “for my well-being” were asked to check from a setlist the effects on their well-being which they expected to obtain from using CBD (multiple responses possible) including improved sleep, reduced anxiety/depression, increased concentration, headache relief, diminished stress, fewer cutaneous problems, increased energy, and reduced pain or inflammation. No questions related to the general health status of the participants or psychiatric disorders were asked.

The study group comprised respondents who reported using CBD in the previous 30 days and who answered the survey question about their primary reason for using CBD. Statistics related to patterns of CBD use for the whole sample were described. We performed logistic regressions to identify the correlates of CBD use, both for well-being (whole sample) as the primary reason and for the specific expected effects listed above. Only variables with a liberal *p*-value < 0.20 (Wald test) in the univariable analyses were considered eligible for the multivariable models. The final multivariable models were built using a backward procedure, and the likelihood ratio test (*p* < .05) was used to define the variables to be maintained in the final models. The Stata/SE 14.2 software (StataCorp LP, College Station, TX) was used for all analyses.

## Results

The sample size included 1166 CBD users. Almost all lived in France (98%). The sample was predominantly male (70%), with a median age of 36 (interquartile range 28–44) years (Table 1). The four most-cited primary reasons to use CBD were “for my well-being” (27%), “to heal my disease or reduce associated symptoms” (25%), “to reduce the use of tobacco or other substances” (12%), and “because I had difficulties obtaining regular (i.e., illicit) cannabis” (9%).

**Table 1:** Sociodemographic and behavioral characteristics of cannabidiol users (*n* = 1166) and logistic regressions of cannabidiol users’ expected effects as outcomes in those who declared “well-being” to be the primary reason for using cannabidiol (*n* = 311)

Variable	Whole sample (*n* = 1166), *N* (%)	Users declaring well-being as the primary reason for cannabidiol use (*n* = 311), *N* (%)	Improved sleep (*n* = 306)			Reduced anxiety/depression (*n* = 306)			Increased concentration (*n* = 290)			Headache relief (*n* = 308)		
			aOR	95% CI	*p*-value	aOR	95% CI	*p*-value	aOR	95% CI	*p*-value	aOR	95% CI	*p*-value
**Sex**														
Male	799 (69.5)	218 (71.2)	–			–			–			–		
Female	351 (30.5)	88 (28.8)	–			–			–			–		
**Age (years)**	36 [28–44]	36 [29–42]	–			–			–			0.96	[0.93–0.99]	0.011
**Smoking tobacco cigarettes in the previous 30 days**														
No	442 (38.4)	130 (42.2)	–			–			–			1		
Yes	631 (54.8)	143 (46.4)	–			–			–			0.48	[0.26–0.89]	0.021
Smoking mainly e-cigarettes	79 (6.9)	35 (11.4)	–			–			–			0.52	[0.18–1.46]	0.215
**Alcohol consumption in the previous 30 days**														
No	346 (30.2)	88 (28.8)	–			–			1			–		
Yes	799 (69.8)	218 (71.2)	–			–			0.49	[0.26–0.91]	0.025	–		
**Illegal cannabis use in the previous 30 days**														
No			–			1			1			–		
Yes			–			0.44	[0.27–0.72]	0.001	1.99	[1.09–3.63]	0.026	–		
**Education level higher than a secondary school diploma**														
No	371 (33.1)	92 (30.9)	–			–			1			–		
Yes	749 (66.9)	206 (69.1)	–			–			2.6	[1.24–5.43]	0.011	–		
**Employment situation**														
Active employment	820 (70.8)	234 (75.5)	–			–			–			–		
Not in active employment	339 (29.2)	76 (24.5)	–			–			–			–		
**Self-reported income level**^a^														
Below average	381 (32.7)	72 (23.2)	1			1			–			–		
Average	545 (46.7)	137 (44.1)	0.75	[0.44–1.30]	0.307	0.42	[0.24–0.73]	0.002	–			–		
Above average	240 (20.6)	102 (32.8)	0.53	[0.28–0.99]	0.049	0.37	[0.19–0.7]	0.003	–			–		
**Body mass index**^b^														
< 25	757 (65.6)	223 (72.9)	1			–			–			–		
≥ 25	397 (34.4)	83 (27.1)	1.8	[1.07–3.01]	0.026	–			–			–		

^a^Income level was self-reported as subjectively assessed as compared to an “average level” estimated by the participants

^b^Body mass index was calculated as the body weight (in kg) divided by the squared height (in m). A body mass index over 25 kg/m^2^ denotes overweight or obesity

In the sub-population which answered well-being (*n* = 311), the effects of CBD most expected by respondents were diminished stress (63% of that group), followed by improved sleep (60%), reduced anxiety/depression (43%), reduced pain or inflammation (41%), increased concentration (16%), and headache relief (16%). Over half the study sample (56%) had first used CBD more than a year prior to the survey, and half (50%) had used it at least 20 of the previous 30 days (Table 2). The most common purchase locations were on the Internet (66%) and in specialized shops (20%). The most common modes of use were smoking CBD-rich cannabis (61%) and ingesting CBD oil sublingually (19%).

**Table 2:** Pattern of cannabidiol use in the whole study sample (*n* = 1166)

	Number	Percent
Time since first CBD use (months)		
Less than 3	260	22.5
Between 3 and 12	255	22.1
Between 12 and 24	308	26.7
More than 24	331	28.7
Number of days CBD used in previous 30 days (days)		
1–3	201	17.2
4–9	166	14.2
10–19	217	18.6
20–29	118	10.1
30 (i.e., every day)	464	39.8
CBD purchase locations in the previous 30 days		
On the internet	769	66.0
Specialized shop	231	19.8
From an acquaintance	56	4.8
Others	51	4.4
Self-cultivation	43	3.7
Tobacco shop	16	1.4
Principal mode of CBD use in the previous 30 days		
Smoking	701	60.7
Sublingual oil	214	18.5
Inhaling	122	10.6
Others (e-liquid, foodstuff, etc.)	117	10.1

*CBD* cannabidiol

In the multivariable analysis performed on the whole sample (Table 1), in terms of tobacco cigarette smoking patterns and the likelihood of reporting well-being as the primary reason to use CBD, smokers were 27% less likely to report it (adjusted odds ratio (aOR) 95% confidence interval (CI): 0.73 [0.55–0.97], *p* = 0.031) than non-smokers, while e-cigarette users were 97% more likely to report it (1.97 [1.2–3.24], *p* = 0.007). Moreover, individuals who smoked illegal cannabis in the previous 30 days (0.73 [0.56–0.95], *p* = 0.021) and those who were actively employed (0.73 [0.54–0.99], *p* = 0.043) were less likely to report well-being as the primary reason. Neither gender nor age was associated with this outcome.

Among those who reported well-being as their primary reason to use CBD, improved sleep was more frequently reported by individuals with lower self-reported income levels and those who were overweight, while individuals who did not use illegal cannabis and those with lower self-reported income levels were more likely to report reduced anxiety/depression as a specific expected effect of using CBD. Alcohol abstinence, illegal cannabis use, and a higher educational level (> secondary school diploma) were all associated with the desire for increased concentration. Finally, younger people and those who did not smoke tobacco cigarettes were more likely to use CBD for headache relief (Table 1). No characteristic was associated with the following specific reasons to use CBD: diminished stress, fewer cutaneous problems, increased energy, and pain or inflammation relief.

## Discussion

Laws governing CBD in the UK are complex and vague, translating into a largely unregulated sector (Gibbs et al., 2019). In France, despite a European Union Court of Justice ruling during the survey period stating that CBD is not a narcotic drug (Court of Justice of the European Union, 2020), its status remains unclear (Bisiou, 2021). Consistent with Moltke et al.’s findings, we found that well-being was one of the most-cited primary reasons for using CBD in our French sample. Additionally, among those who declared this, reduced anxiety, improved sleep, and diminished stress were the expected effects most cited. Unlike Moltke et al., gender was not associated with any reason for using CBD. Our French study population differed from the UK one in several ways: the male-female ratio was higher, time since first CBD use was longer, and a larger proportion of participants smoked CBD. While these differences may partly be due to recruitment methods, country-related specificities cannot be excluded. For example, law enforcement policies in both countries seem to differ. In the UK, police preferentially target CBD in herbal form, while no such distinction between CBD products is made in France. This may partly explain the lower preference for smoking CBD in UK users than in our French sample.

To conclude, in our French user sample from the general population, we duplicated Moltke et al.’s findings for UK users that well-being, sleep, stress, and anxiety are the main reasons for using CBD. However, factors associated with reasons for use differed between both studies as did patterns of use. Unlike Moltke et al., we did not find any gender effect. We agree with their conclusion that further research is needed to clarify whether CBD is effective in treating the health problems studied, and we also call for more research to highlight and better understand country-specific patterns of CBD use and correlates of use.

## Data Availability

The datasets generated and/or analyzed during the current study are not publicly available due to ongoing data treatment but are available from the corresponding author on reasonable request.
